# Both common and specialty mushrooms inhibit adhesion molecule expression and in vitro binding of monocytes to human aortic endothelial cells in a pro-inflammatory environment

**DOI:** 10.1186/1475-2891-9-29

**Published:** 2010-07-16

**Authors:** Keith R Martin

**Affiliations:** 1 Healthy Lifestyles Research Center, College of Nursing and Health Innovation, Arizona State University, 6950 East Williams Field Road, Mesa, AZ 85212, USA

## Abstract

**Background:**

Cardiovascular disease (CVD) is a leading cause of mortality in the United States as well as globally. Epidemiological studies show that regular fruit and vegetable consumption reduces CVD risk, in part, due to antioxidant activity and immunomodulation since oxidative stress and inflammation are features of atherogenesis. Accumulating evidence also shows that dietary fungi, viz., mushrooms, can protect against chronic disease by altering inflammatory environments such as those associated with CVD although most research has focused on specialty mushrooms. In this study, we tested the ability of both common and specialty mushrooms to inhibit cellular processes associated with CVD.

**Methods:**

Human aortic endothelial cells (HAEC) were incubated overnight with control media with dimethylsulfoxide (DMSO) vehicle (1% v/v) or containing DMSO extracts of whole dehydrated mushrooms (0.1 mg/mL), which included *Agaricus bisporus *(white button and crimini), *Lentinula edodes *(shiitake), *Pleurotus ostreatus *(oyster), and *Grifola frondosa *(maitake). Monolayers were subsequently washed and incubated with medium alone or containing the pro-inflammatory cytokine IL-1β (5 ng/mL) for 6 h to upregulate pro-atherosclerotic adhesion molecules (AM). AM expression was assayed by ELISA and binding of U937 human monocytes pre-loaded with fluorescent dye was determined.

**Results:**

White button mushrooms consistently reduced (p < 0.05) VCAM-1, ICAM-1, and E-selectin-1 expression, whereas other test mushrooms significantly modulated AM expression singly, collectively, or combinatorially. All mushrooms, however, significantly reduced binding of monocytes to both quiescent and cytokine-stimulated monolayers.

**Conclusion:**

These data provide evidence that dietary mushrooms can inhibit cellular processes such as adhesion molecule expression and ultimate binding of monocytes to the endothelium under pro-inflammatory conditions, which are associated with CVD. As a result, these findings support the notion that dietary mushrooms can be protective against CVD.

## Background

Cardiovascular disease (CVD) is a leading cause of morbidity and mortality in the United States as well as globally in both developed and developing countries [1]. Epidemiological studies show that regular consumption of plants, i.e., fruits and vegetables, is strongly and convincingly associated with a reduced risk of chronic disease including CVD [2,3]. This protection presumably occurs due to a plethora of bioactive phytochemicals that can modulate processes including the immune response, inflammation and antioxidant activity [4,5]. In addition to plants, dietary fungi, viz., mushrooms, also contain a diverse array of biologically active molecules rendering them potentially protective against CVD [6,7]. In fact, dietary mushrooms have been shown in previous studies to improve cardiovascular health, stimulate immune function, contribute to glucose homeostasis, and to modulate detoxification, as well as exert anti-allergic, anti-tumor, anti-viral, antibacterial, antifungal, and anti-inflammatory activities [5,8-10]. As a result, both cellular components and secondary metabolites of myriad dietary mushrooms have been used in treatment for a variety of diseases [11]. While previous results have been compelling, research has largely focused on specialty or exotic mushrooms associated with the Far East including shiitake, maitake, and reishi. However, the white button mushroom is the most frequently consumed mushroom in the United States and could be equally effective in preventing or slowing CVD [10].

The etiology of CVD involves, in part, a complex process of development and deposition of cholesterol-ladened fatty streaks within aortic blood vessels and appears associated with oxidative stress and inflammation [12,13]. Accumulating evidence suggests also a critical link between inflammation and metabolic syndrome, CVD, and diabetes [14]. Pro-inflammatory cytokines such as IL-1β, chemokines, and upregulation of several key adhesion molecules including intercellular adhesion molecule-1 (ICAM-1), vascular cell adhesion molecule-1 (VCAM-1), and endothelial-leukocyte adhesion molecule-1 (ELAM-1 or E-selectin) have been shown to contribute significantly to CVD by initiating an interaction between the vascular endothelium and monocytes, precursors to foam-ladened macrophages [13,15]. The increased expression of adhesion molecules, migration of monocytes into the aortic subendothelium, and foam cell formation can contribute to atherosclerotic plaque development and premature cardiovascular disease and death. It has been demonstrated that an atherogenic diet high in dietary fat such as the Western diet can also rapidly induce adhesion molecules and contribute to atherogenesis [16]. Some dietary agents, such as mushrooms, can inhibit or attenuate these processes, which would be beneficial in slowing or preventing downstream chronic disease.

Despite emerging evidence, little attention has been focused on the potential protective role of edible mushrooms against atherogenesis in a biological context or determination of a specific underlying mechanism. Moreover, a lingering question is whether common dietary mushrooms can be as effective as specialty, or exotic, mushrooms. In this research, we have determined whether both common mushrooms and specialty mushrooms can modulate critical events leading to atherogenesis such as pro-inflammatory, cytokine-induced upregulation of adhesion molecule expression to include VCAM-1, ICAM-1, and E-selectin. We selected as test mushrooms *Agaricus bisporus *(white button and crimini varieties), *Lentinula edodes *(shiitake), *Pleurotus osteratus *(oyster), and *Grifola frondosa *(maitake). After analyzing VCAM-1, ICAM-1, and E-selectin expression, we assessed whether significant reductions in binding of human monocytes to aortic endothelium in a quiescent or a pro-inflammatory environment occurred. We hypothesized that preincubation of human aortic endothelial cells (HAEC) with dimethylsulfoxide (DMSO) extracts of whole mushrooms would inhibit the adverse binding of U937 human monocytes to cytokine-stimulated aortic endothelium using an *in vitro *model of atherogenesis.

## Methods

### Preparation and delivery of mushrooms

Samples of mushrooms were collected from The Pennsylvania State University Mushroom Test Demonstration Facility and Mushroom Research Center and Modern Mushroom Farm, Inc. (Toughkennamon, PA) as outlined in Table 1. Mushrooms were grown using the standard tray system under controlled conditions and were harvested at the optimum maturation stage with closed caps that were 2.0-2.5 inches in diameter [17]. Mushrooms from the second break of the *A. bisporus *crops were tested and included brown (crimini) mushrooms and the common white button mushrooms. Specialty mushrooms were harvested using standard mycology protocols and harvested on peak production days [17].

**Table 1:** Mushrooms selected for testing

Test Mushroom	Genus/Species	Sample type	Source
Shiitake	*Lentinula edodes*	Basidioma	Modern Mushroom Farm
Crimini	*Agaricus bisporus*	brown mushroom	Penn State University
Oyster	*Pleurotus ostreatus*	Basidioma	Modern Mushroom Farm
Maitake	*Grifola frondosa*	Basidioma	Modern Mushroom Farm
White Button	*Agaricus bisporus*	all crops	Penn State University

Harvested mushroom crops were randomly sampled, cleaned, sliced, and stored at 0°C for 24 h. Samples were later freeze-dried (Model 15 SRC-X; Virtis Genesis Co, Inc., Gardiner, NY), ground to a fine powder, and sieved through a 16 mesh screen. Mushroom powders were collected in sterile sample bags (Fisher Scientific, Pittsburgh, PA) and stored in the dark at room temperature in desiccators prior to analysis.

After analyses, lyophilized mushroom powders were stored desiccated at -80°C in the dark until use. Stock solutions of each test mushroom were prepared by dissolving 100 mg mushroom powder into 10 mL DMSO (Sigma, St. Louis, MO) in tubes immersed in ice followed by three cycles of 2 min each of sonication using a Bransonic sonifier (Model S450, Danbury, CT). Immediately prior to experiments, aliquots of stock solutions (0.01 mL) were independently diluted into endothelial cell basal medium (EBM) medium to produce 0.1 mg/mL working concentrations each with 1% (v/v) DMSO. DMSO was not toxic at this concentration as determined by viability assays when compared to control cultures with medium alone (data not shown).

### Cell culture

HAEC were purchased from Clonetics Laboratories (San Diego, CA) and cultured in EBM (Clonetics, San Diego, CA). The medium contained 10% fetal bovine serum (FBS), hydrocortisone, vascular endothelial growth factor, insulin-like growth factor, fibroblast growth factor, gentamicin, epidermal growth factor, heparin, and ascorbic acid at concentrations provided as a kit by the manufacturer (Clonetics, San Diego, CA). HAEC were seeded in 1% gelatin (Sigma, St. Louis, MO) coated Corning T-75 flasks, 24, and 96-well plates (Corning, NY). Medium was changed every other day and cells were subcultured by trypsinization, resuspension, and reseeding to flasks to propagate cultures. For experiments, HAEC were used for three passages (number of times subcultured) since they are non-transformed and non-immortalized. For this study, cells were grown to confluence and each assay was replicated 3-4 times in quadruplicate using passages 6-8. U-937 monocytes (ATCC, Rockville, MD) grew in suspension culture in RPMI-1640 medium (Sigma, St. Louis, MO) supplemented with 10% FBS, 2 mM glutamine, 100 U/mL penicillin, and 100 ug/mL streptomycin. Medium was exchanged every 3 d and cells were subcultured weekly.

### Cytotoxicity of mushroom powders

Monolayers of HAEC were incubated overnight with media containing DMSO vehicle alone (1% v/v) or containing the test mushroom powders. Cytotoxicity was determined by changes in morphology as determined by light microscopy and trypan blue exclusion as an indicator of viability.

### Adhesion molecule expression

HAEC were cultured in 24-well plates until confluent and were incubated overnight with mushroom powders dissolved in cell culture medium as described above. After incubation, monolayers were washed twice with phosphate buffered saline (PBS) (0.5 mL/well) and further incubated with medium alone or with IL-1β (5 ng/mL) for 6 h at 37°C. Medium was removed and monolayers were fixed with 1% formaldehyde at 25°C for 30 min. Monolayers were washed with PBS and blocked with 10% FBS in PBS for 1 h to reduce non-specific binding and background. Monoclonal antibodies against human VCAM-1, ICAM-1, and E-selectin (BD Pharmingen, San Diego, CA) were added at 5, 1, and 10 μg/mL, respectively, in PBS with 10% FBS for 1 h at 25°C. The secondary antibody, horseradish-conjugated anti-mouse IgG (Santa Cruz, CA), was added at a 1:400 dilution and incubated at 25°C for 2 h. Subsequently and after washing with PBS, horseradish peroxidase substrate was added and cells were incubated for 1 h to develop the colorimetric endpoint (Bio-Rad, Hercules, CA). The optical densities, or absorbances, were read at 405 nm using a Tecan SpectraFluor multi-well plate reader (Tecan, Research Triangle Park, NC).

### Fluorescent labeling of monocytes

U-937 cells (ATCC, Rockville, MD), a human monocyte cell line, was used to test adherence to HAEC monolayer [18]. Prior to co-incubation, U-937 cells were fluorescently labeled by incubating cells (2 × 10^6 ^cell/mL) with 5 μM BCECF-AM/L [2', 7'-bis-(2-carboxyethyl)-5 (and 6)-carboxy-fluorescent acetoxymethyl ester] (Molecular Probes, Eugene, OR) in EBM medium for 30 min at 37°C and 5% CO_2 _as described previously [19]. BCECF-AM, a non-fluorescent lipophilic compound, readily enters cells where it is modified to become fluorescent and retained by cells. BCECF-AM was prepared as a 1 mg/mL stock in DMSO and stored at -80°C. After labeling, cells were washed twice with PBS containing 1% FBS to remove excess dye. U937 cells were then resuspended in medium (5 × 10^5 ^cells/mL) and added (0.5 mL/well) to HAEC monolayers as described below.

### U-937 monocyte binding to HAEC

HAEC were cultured in gel-coated 24-well plates until confluent then incubated overnight with mushroom powders dissolved in cell culture medium as described above. After incubation, monolayers were washed twice with PBS (0.5 mL/well) and further incubated with IL-1β (5 ng/mL) for 6 h at 37°C. U-937 cells, labeled as described above, were incubated with HAEC for 30 min at 37°C and 5% CO_2_. After incubation, non-adherent cells were removed by washing twice with PBS containing 1% FBS. The attached cells were lysed with 0.5 mL of 50 mM Tris buffer (pH 7.6) containing 1% sodium dodecyl sulfate (SDS). The fluorescent intensity was measured at excitation and emission wavelengths of 485 and 535 nm, respectively, with a Tecan Spectrafluor fluoresence multiwell plate reader.

### Statistical analysis

Data were analyzed using the Students t test to compare individual treatment groups with the respective controls. Values are displayed as means + SEM and p < 0.05 is considered significant.

## Results

### VCAM-1 expression

**Figure 1:**
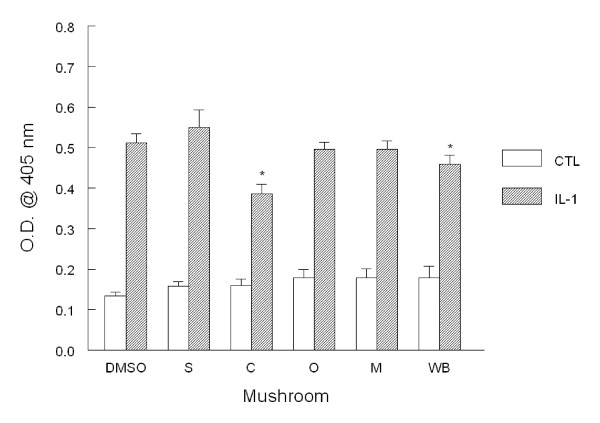
**VCAM-1 adhesion molecule expression in HAEC**. Monolayers were incubated overnight with 1% (v/v) DMSO alone (DMSO) or with mushroom powder (0.1 mg/mL) including shiitake (S), crimini (C), oyster (O), maitake (M), and white button (WB). Cell monolayers were washed then incubated with medium alone or IL-1β (5 ng/mL) for 6 h. After harvesting, VCAM-1 expression was determined by ELISA. Data indicate mean optical density (O.D.) ± SEM and are representative of 3-4 experiments each performed in quadruplicate. *p < 0.05 vs. control.

Increased expression of adhesion molecules and binding of monocytes to HAEC are critical events in atherogenesis. As a result, we first analyzed the expression of VCAM-1 after pre-incubation with medium alone or containing mushroom powder. After stimulation with IL-1β for 6 h, VCAM-1 expression significantly increased by 3.8 fold in DMSO vehicle control cells (Fig. 1). There were no differences between stimulated DMSO vehicle-treated cells and stimulated cells incubated with medium alone. After preincubation with mushrooms, VCAM-1 expression was marginally, but significantly, reduced by 24.8 amd 10.5% after crimini and white button mushroom powder, respectively. Basal expression of VCAM-1 was not significantly altered after pre-incubation with mushrooms.

### ICAM-1 expression

**Figure 2:**
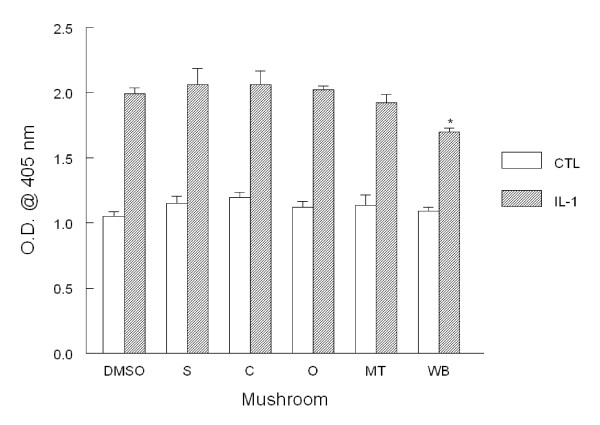
**ICAM-1 adhesion molecule expression in HAEC**. Monolayers were incubated overnight with 1% (v/v) DMSO alone (DMSO) or with mushroom powder (0.1 mg/mL) including shiitake (S), crimini (C), oyster (O), maitake (M), and white button (WB). Cell monolayers were then washed and incubated with medium alone or IL-1β (5 ng/mL) for 6 h. After harvesting, ICAM-1 expression was determined by ELISA. Data indicate mean optical density (O.D.) ± SEM and are respresentative of 3-4 experiments each performed in quadruplicate. *p < 0.05 vs. control.

We next analyzed the expression of the adhesion molecule ICAM-1. Basal ICAM-1 expression was higher than either basal VCAM-1 or E-selectin as observed previously and routinely and was not altered by pre-incubation with solutions from mushroom powder. Incubation with IL-1β increased significantly ICAM-1 expression by 2-fold as expected (Fig. 2). After pre-incubating with mushroom extracts, ICAM-1 expression was marginally, but significantly, decreased by white button mushroom powder by 8%. Other test mushrooms did not significantly reduce ICAM-1 adhesion molecule expression.

### E-selectin expression

**Figure 3:**
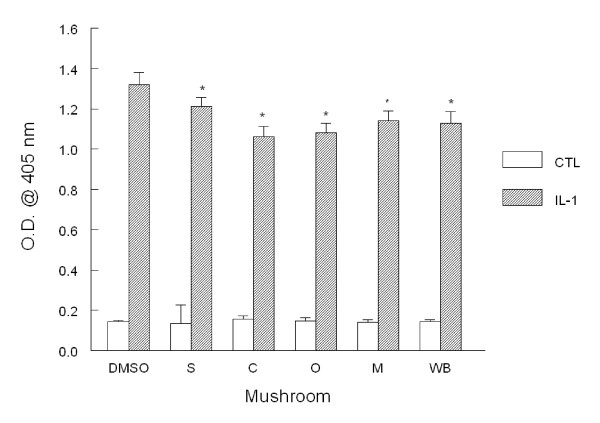
**E-selectin adhesion molecule expression in HAEC**. Monolayers were incubated overnight with 1% (v/v) DMSO alone (DMSO) or with mushroom powder (0.1 mg/mL) including shiitake (S), crimini (C), oyster (O), maitake (M), and white button (WB). Cell monolayers were washed then incubated with medium alone or IL-1β (5 ng/mL) for 6 h. After harvesting, E-selectin expression was determined by ELISA. Data indicate mean optical density (O.D.) ± SEM and are representative of 3-4 experiments each performed in quadruplicate. *p < 0.05 vs. control.

We next analyzed the expression of the adhesion molecule E-selectin. Basal expression remained low and unchanged in all cultures including treatment groups (Fig. 3). After incubation with IL-1β, E-selectin expression significantly increased by 9.2-fold. After pre-incubation with mushrooms, E-selectin expression was significantly reduced by 8, 20, 18, 13, and 14% after pre-incubation with shiitake, crimini, oyster, maitake, and white button mushroom powders, respectively.

### Binding of U937 monocytes

**Figure 4:**
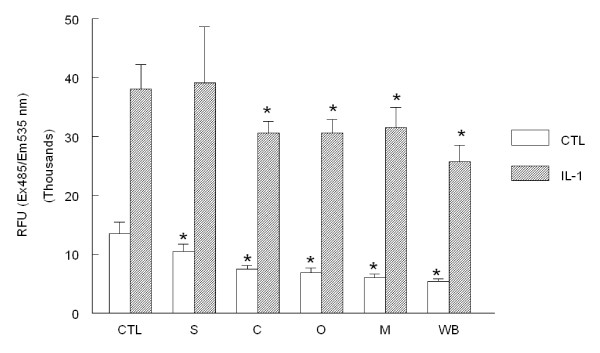
**Binding of U937 human monocytes to HAEC**. HAEC monolayers were incubated overnight with 1% (v/v) DMSO alone (DMSO) or with mushroom powder (0.1 mg/mL) including shiitake (S), crimini (C), oyster (O), maitake (M), and white button (WB). Monolayers were washed then incubated with medium alone or containing IL-1β (5 ng/mL) for 6 h. After washing again to remove IL-1β, fluorescently labeled U937 cells were co-incubated with HAEC for 30 min at 37°C. HAEC monolayers and bound U937 cells were harvested and the total fluorescence was analyzed to determine monocyte binding. Data indicate mean relative fluorescent units (RFU) ± SEM and are resentative of 3-4 experiments each performed in triplicate. *p < 0.05 vs. control.

We next analyzed the capacity for pre-loaded U937 monocytes to bind and adhere to human aortic endothelial cells. Pre-incubation with mushrooms reduced adhesion of monocytes to unstimulated HAEC significantly by 23, 44, 50, 55, and 60% for shiitake, crimini, oyster, maitake, and white button mushroom powders, respectively (Fig. 4). Incubation with IL-1β significantly increased monocyte adherence to HAEC by 2.7-fold (Fig. 4). Pre-incubation with crimini, oyster, maitake, and white button significantly reduced U937 adhesion to IL-1β-stimulated HAEC by 18, 18, 16, and 26%, respectively. Pre-incubation with shiitake was not different from control cultures and did not affect binding of monocytes.

## Discussion

This study is the first to demonstrate the capacity of whole mushrooms, delivered as DMSO extracts, to reduce adhesion molecule expression with subsequent reductions in human monocyte binding to human aortic endothelial cells. Specifically, we have demonstrated that dietary mushrooms significantly reduced cell surface expression of VCAM-1, ICAM-1, and E-selectin. Furthermore, there were significant reductions in adhesion of pre-loaded human monocytes to both unstimulated, or quiescent, and pro-inflammatory, cytokine-stimulated HAEC as summarized in Table 2. The health implications are that diverse mushrooms, including common and specialty mushrooms can protect against cardiovascular disease by interfering with events that contribute to atherogenesis.

**Table 2:** Summary of effects by mushrooms on adhesion molecule expression and monocyte binding

Mushroom	VCAM-1	ICAM-1	E-selectin	Binding-U*	%	Binding-S	%
Shiitake			reduced	reduced	23	no change	0
Crimini	reduced		reduced	reduced	44	reduced	18
Oyster			reduced	reduced	50	reduced	18
Maitake			reduced	reduced	55	reduced	16
White button	reduced	reduced	reduced	reduced	60	reduced	26

*U, unstimulated HAEC cells; S, stimulated with IL-1β

Mushrooms have received marginal attention regarding their potential role in protecting against cardiovascular disease although studies have suggested a protective effect against CVD. For example, powders (5% dry weight) of shiitake, maitake, and *Agaricus bisporus *significantly decreased plasma cholesterol levels and blood pressure in rats presumably due to the bioactive agent eritadenine [20-22]. Furthermore, dietary oyster mushrooms (*P. ostreatum*) also elicited a hypocholesterolemic effect and inhibited lipid peroxidation, a process linked to oxidative stress, in rabbits [23,24]. This is in agreement with our results in that white button and crimini (*A. bisporus*), maitake and oyster significantly reduced AM expression and monocyte binding, which would counter a hypercholesterolemic, or hyperlipidemic, effect. That is, even with elevated plasma levels of lipids, the reduced expression of AM and subsequent inhibition of binding of monocytes would interrupt the atherogenic process. This is further supported by the observation that a 10% (w/w) oyster mushroom diet significantly reduced both the incidence and size of aortic atherosclerotic plaques in rabbits suggesting interaction with physiological and molecular processes of atherogenesis [23,24]. Percario et al. also showed that a natural antioxidant-rich mushroom, *Agaricus sylvaticus*, could prevent the development of atherosclerosis in an environment of hypercholesterolemia using a rabbit model [25]. Collectively, several studies suggest that dietary mushrooms can inhibit or slow CVD although more research is needed.

In this report, we have demonstrated that both common and specialty mushrooms can modulate processes at the cellular level where AM expression is attenuated and binding of monocytes blunted. Others have identified alternative mechanisms for a potential CVD protective effect by dietary mushrooms. For example, oyster mushrooms (10% of diet) reduced significantly the incidence of atherosclerotic plaques in rabbits as well as plaque size by 26% compared to the control group and prevented formation of atheromas, reduced foam cell number, and reduced coronary arterial injury [24]. Other dietary antioxidants (vitamins C, E, and beta carotene) have been shown to reduce lesion area by reducing concentrations of soluble adhesion molecules by 36-61% in mice [26]. This observation is important because soluble AM have been proposed as a critical assessment tool for asymptomatic CVD in high-risk patients [27]. While we did not measure soluble AM in this study, the capacity of the test mushrooms to reduce cell surface expression in HAEC is equally compelling as a protective mechanism. Other proposed mechanisms of cardioprotection in rodents consuming *Grifola frondosa*, *Inonotus obliquus*, *Antrodia xantha*, and *Rigidoporus ulmarius *include inhibition of COX-1 and COX-2 activities, inhibition of iNOS via reduced NF-kB binding, and potent antiangiogenesis activity [28-30]. Collectively, the data support the capacity of both exotic and commonly consumed mushrooms to beneficially modulate numerous processes associated with atherogenesis.

We also noted in this study that the three most frequently measured AM including VCAM-1, ICAM-1 and E-selectin, were reduced singly, collectively, or in various combinations of two after incubation with mushrooms yet binding of monocytes was consistently reduced after incubation with each mushroom extract. Others have shown similar results with different dietary agents using the HAEC cell model, which is a non-transformed primary endothelial cell line. For example, pretreatment of HAEC with a red wine polyphenol extract significantly inhibited oxysterol-induced cell surface expression of adhesion molecules and subsequent adherence of monocytes to monolayers by inhibiting primarily VCAM-1 [31]. Subsequent studies by Naito et al. showed that tocotrienols exerted a similar effect [32]. Dietary consumption of antioxidant polyphenols from avenathramide-enriched mixtures (AEM) derived from oats significantly reduced IL-1β-stimulated VCAM-1, ICAM-1, and E-selectin expression in HAEC by 50, 20, and 20% respectively, and reduced U937 binding at concentrations as low as 20 μg/mL [33]. Ginkgo biloba extracts reduced both VCAM-1 and ICAM-1 and adhesion of monocytes to HAEC [34]. In a study using structurally related dietary carotenoids, Martin et al. found that pre-incubation of HAEC with beta-carotene, lutein and lycopene significantly reduced VCAM-1 expression, beta-carotene and lutein significantly reduced E-selectin expression, and beta-carotene, lutein and lycopene significantly reduced the expression of ICAM-1, yet lycopene was the only carotenoid to significantly reduce binding of U937 to HAEC [19]. The antioxidant pyrrolidine dithiocarbamate selectively inhibited in HUVEC cells VCAM-1 induction, but not ICAM-1, after stimulation with TNF-α and LPS. E-selectin was only partially inhibited after TNF-α incubation [35]. Pretreatment of HUVECs with vitamin E and probucol significantly reduced the expression of VCAM-1 induced by oxidized LDL, but did not reduce expression of ICAM-1 [36]. In contrast, the well recognized antioxidants N-acetyl-L-cysteine and troglitazone, an antidiabetic agent, reduced or completely abolished VCAM-1, E-selectin, and ICAM-1 expression in endothelial cells after pro-oxidant signals including oxidized LDL and TNF-α [37]. Thus, each of the three commonly tested AM can be modulated either singly or in combination by diverse dietary agents and typically at least one, but often more, are inhibited. This suggests that attenuation of at least one AM, without specificity as to which one, may be adequate for inhibition of binding of monocytes but also that there are many other contributors to this process that may not be measured.

It is noteworthy that mushrooms also contain high levels of bioactive agents including polyphenols and the novel antioxidant ergothioneine, which is produced exclusively in mushrooms and some bacteria [38,39]. Dietary polyphenols such as catechin and quercetin have been shown to significantly reduce VCAM-1 expression and binding of monocytes to HAEC and ergothioneine exerts antioxidant activity that may be protective in a pro-inflammatory environment [40-42]. The test mushrooms used in this study have been analyzed and found to contain considerable amounts of both ergothioneine and total polyphenols [43,44]. Moreover, all test mushrooms exhibited significant antioxidant capacities using assays for oxygen radical absorbance capacity (ORAC), and hydroxyl (HORAC), peroxynitrite (NORAC), and superoxide (SORAC) radical averting capacity. Mushrooms also contain high levels of selenium and copper, essential micronutrients, needed for proper function of antioxidant enzymes, which can reduce oxidative stress and inflammation often found in CVD [45]. It is possible that varying levels of these bioactive agents alone or in combination contributed to the beneficial effects observed.

## Conclusions

In conclusion, dietary mushrooms sampled from both common, which are routinely consumed in the United States, and specialty mushrooms interfered with processes critical to atherogenesis and cardiovascular disease. These findings further support the notion that consumption of not only fruits and vegetables, but also dietary fungi, viz., mushrooms, is an important approach to minimizing CVD risk. Moreover, common, readily available and affordable mushrooms such as white button, or *Agaricus bisporus*, as well as specialty mushrooms including shiitake appear particularly beneficial to health.

## Competing interests

The author declares that they have no competing interests.

## Authors' contributions

KRM conceived and designed the study, performed the experiments, analyzed samples, gathered data, conducted statistical analyses, and prepared the draft manuscript. The author has read and approved the final manuscript.
